# Mupirocin-Resistant *Staphylococcus aureus* in Iran: A Biofilm Production and Genetic Characteristics

**DOI:** 10.1155/2022/7408029

**Published:** 2022-01-15

**Authors:** Samira Zamani, Anis Mohammadi, Bahareh Hajikhani, Parnaz Abiri, Maryam Fazeli, Mohammad Javad Nasiri, Masoud Dadashi, Mehdi Goudarzi, Mehrdad Haghighi

**Affiliations:** ^1^Department of Microbiology, School of Medicine, Shahid Beheshti University of Medical Sciences, Tehran, Iran; ^2^Department of Microbiology, Faculty of Advanced Science and Technology, Tehran Medical Sciences, Islamic Azad University, Tehran, Iran; ^3^Department of Microbiology, Faculty of Biological Sciences, North Tehran Branch, Islamic Azad University, Tehran, Iran; ^4^Deputy of Research, Faculty of Medicine, Hamadan University of Medical Sciences, Hamadan, Iran; ^5^Department of Microbiology, School of Medicine, Alborz University of Medical Sciences, Karaj, Iran; ^6^Department of Infectious Diseases, Imam Hossein Teaching and Medical Hospital, Shahid Beheshti University of Medical Sciences, Tehran, Iran

## Abstract

The spread of mupirocin-resistant *Staphylococcus aureus* strains in hospitals and communities is a universal challenge. Limited data is available on the genetic features of high-level mupirocin resistant- (HLMUPR-) *S. aureus* isolates in Tehran. In the present research, we investigated 48 high-level mupirocin resistance *S. aureus* by antimicrobial activity, virulence analysis, biofilm formation, multilocus sequence typing (MLST), and staphylocoagulase (SC) typing. All the HLMUPR strains were positive for *mupA* gene. The frequency of multidrug resistance was 97.9%. Twenty-one (43.8%) were toxinogenic with 14 producing *pvl* (29.2%), 5 *tst* (10.4%), and two *eta* (4.2%). Among the HLMUPR isolates, biofilm production was detected in 45 (89.6%) isolates with complete dominance *clfB*, *clfA* genes, and a noticeably high frequency *fnbA* (95.8%), followed by *fnbB* (93.8%), *eno* and *icaD* (each 83.3%), *sdrC* (81.3%), *ebps* (79.2%), *icaA* (75%), *sdrD* (66.7%), *fib* (60.4%), *sdrE* (50%), *cna* (41.7%), and *bap* (4.2%). Coagulase typing distinguished isolates into four genotypic patterns including III (50%), II (27.1%), and type IVa (22.9%). A total of three clonal complexes (CCs) and 4 sequence types (STs) including CC/ST22 as the most prevalent (52.1%), CC8/ST239 (20.8%), CC/ST8 (16.7%), and CC/ST5 (10.4%) were identified in current work. According to our analysis, nonbiofilm producer isolates belonged to CC8/ST239 (6.3%) and CC/ST8 (4.2%). Fusidic acid-resistant isolates belonged to CC/ST45 (*n* = 3) and CC8/ST239 (*n* = 1). Observations highlighted the circulation of the CC/ST22 HLMUPR *S. aureus* strains with strong biofilm-production ability in our hospitals, indicating the possibility of transmission of this type between community and hospital.

## 1. Introduction

As the most frequent pathogen, *Staphylococcus aureus* causes various diseases ranging from skin and soft tissue infections (SSTIs) to bacteremia, endocarditis, and osteomyelitis in hospitals and communities. Recent evidence has highlighted a dramatic increase in hospital-acquired *S. aureus* infections worldwide with significant morbidity and mortality [1–3]. The high prevalence of antibiotic resistance due to inappropriate antibiotic use and the remarkable ability of this bacterium to acquire resistance to multiple antimicrobials, particularly mupirocin-resistant *S. aureus* (mupR-*S. aureus*), complicated the treatment of related infections. Moreover, the scenario worsens with resistance to commonly used antibiotics, biofilm-forming ability, and various virulence factors for *S. aureus* [4–6].

Mupirocin is a topical antibiotic with excellent activity in treating SSTIs and eradicating MRSA carriage in nursing homes, healthcare workers, patients, and control of MRSA outbreaks. However, an increase in mupirocin resistance has been reported widely in many areas of the world following its extensive and widespread use [7–10]. Mupirocin, an analog of isoleucyl, interferes with protein synthesis by competitive inhibition of the bacterial isoleucyl-tRNA synthetase (IRS). Resistance to mupirocin has been described in two categories: (i) high level that occurs via plasmid-borne gene *mupA* and or the newly described *mupB* gene, coding for an alternate isoleucyl-tRNA synthetase (IleS), and (ii) low level, which results from point mutations in the chromosomal encoding isoleucyl-tRNA synthetase (*ileS*) gene. Data worldwide indicate that lack of proper and timely identification, distribution of molecular types, and managing of high-level mupirocin resistance *S. aureus* infections (HLMUPR) could lead to decolonization failure, increase in carriage rate, and subsequently wide range of staphylococcal infections [10, 11]. Diagnosis of HLMUPR in *S. aureus* clinical isolates is a great challenge due to the distribution of different types related to the type of infection, their antimicrobial profile, and biofilm formability [10]. On the other hand, timely diagnosis of HLMUPR in *S. aureus* clinical isolates is of great significance for formulating appropriate treatment plans, shortening hospitalization time, and reducing the disease burden [11]. Since extensive studies have not been performed in connection with HLMUPR *S. aureus* infection and the high importance of its timely diagnosis and treatment, therefore, it has become urgently needed to figure out the antibacterial activity, carriage of virulence determinants, biofilm-forming ability, and specific genes that may be involved in biofilm formation and molecular types in HLMUPR strains isolated from various clinical samples. In order to produce a more comprehensive description of the molecular epidemiology and phenotypic properties of HLMUPR *S. aureus* in Iran, we characterized 390 S*. aureus* isolates collected from several geographically dispersed Tehran hospitals over the past two years. The frequency of fourteen selected genes involved in biofilm production was assessed to identify genes involved in biofilm production. Coagulase (*coa*) typing and multilocus sequence typing (MLST) were applied to determine the molecular types of the HLMUPR strains.

## 2. Materials and Methods

### 2.1. Isolation S. aureus, Genomic DNA Extraction, and Identification of HLMUPR Isolates

In the present study, 390 S*. aureus* strains from clinical samples of patients referred to the hospitals affiliated to Shahid Beheshti University of Medical Sciences from January 2018 to December 2019 were screened for HLMUPR. Identification of *S. aureus* was performed by microbiological and biochemical tests and the presence of the *nuc* gene using polymerase chain reaction (PCR) assay [12]. The DNA extraction was performed by the phenol-chloroform extraction method previously described by Goudarzi et al. [12]. The purity of the extracted DNA was evaluated by a NanoDrop-2000 spectrophotometer (Thermo Fisher Scientific, Wilmington, DE, USA) at 260/280, respectively. An A260/A280 purity ratio of ~1.8 was considered for the PCR assay.

The study was ethically approved by the Ethics Committee of the Shahid Beheshti University of Medical Sciences in Tehran, Iran (IR. SBMU. MSP.REC. 1398. 353), and consent was obtained from consent participants. HLMUPR strains were detected by the broth microdilution method (MIC value was ≥512 *μ*g/ml) according to the clinical and the laboratory standards institute (CLSI) guideline (CLSI 2019) and PCR of *mupA* gene [4]. HLMUPR strains that were obtained on or after 96 hours of admission to a hospital were considered as hospital onset (HO) while a positive culture prior to four days of hospitalization along with one or more of the following criteria: (i) a history of hospitalization, surgery, dialysis, or residence in a long-term care facility in 12 months prior to culture date or (ii) the presence of a central vascular catheter (CVC) within two days before *S. aureus* culture was considered as community onset (CO).

### 2.2. In Vitro Antimicrobial Susceptibility of HLMUPR Isolates

The antibiotic resistance pattern of isolates was evaluated using the Kirby Bauer method according to the CLSI instructions against amikacin (AMK), clindamycin (CLI), ciprofloxacin (CIP), erythromycin (ERY), gentamicin (GEN), kanamycin (KAN), linezolid (LIN), penicillin (PEN), quinupristin-dalfopristin (SYN), rifampicin (RIF), tobramycin (TOB), tetracycline (TET), teicoplanin (TEC), and trimethoprim-sulfamethoxazole (SXT) (Mast Co., UK). MRSA strains were identified phenotypically using the Cefoxitin disk diffusion method (30 *μ*g) according to the CLSI guidelines and detection of the *mecA* gene as previously described [4].

The microdilution was performed to determine minimum inhibitory concentration (MIC) titer for antibiotics vancomycin (VAN), tigecycline (TIG), and fusidic acid (FUS) according to the procedure detailed in our previous report [4]. Results for fusidic acid and tigecycline were interpreted based on the European Committee for antimicrobial susceptibility testing (EUCAST) recommendations (http://www.eucast.org). The inducible and constitutive macrolide-lincosamide-streptogramin group B (iMLS_B_ and cMLS_B_) resistance phenotypes were identified by erythromycin and clindamycin disks by the CLSI guideline (CLSI 2019). *S. aureus* ATCC 25923, ATCC 43300, and ATCC 29213 strains were used for antibiotic susceptibility testing quality control.

### 2.3. Microtiter Plate (MtP) Assay to Determine Biofilm Formability

The ability of bacterial biofilm formation was assessed using MtP assay as Yousefi et al. [9] described. All tests were run in quadruplicate and rerun three separate times to confirm reproducibility. *Staphylococcus epidermidis* ATCC 35984 strain was used as a positive control strain for biofilm formation. TSB supplemented with 1% glucose was used as a negative control. Considering an optical density cut-off (ODc) to be represented by average OD of negative control + 3 × standard deviation (SD) of negative control, strains were classified into the following categories: without biofilm (OD ≤ 0.059), weak (ODc < OD ≤ 2 × ODc), moderate (2 × ODc < OD ≤ 4 × ODc), and strong (OD > 4 × ODc) [9].

### 2.4. Screening for Virulence-Related Genes

For determination of the prevalence of Panton-Valentine leukotoxin (*pvl*), toxic shock syndrome toxin (*tsst-1*), and exfoliative toxin (*eta* and *etb*) genes, PCR reaction was carried out with specific oligonucleotide primers [12]. For definitive confirmation of USA300 strains, the existence of the arginine catabolic mobile (ACME) elements in all the isolates was investigated employing PCR assay as previously described by Boswihi et al. [13].

### 2.5. Detection of Adhesion and Biofilm Genes

The existence of the clumping factors A and B (*clfA* and *clfB*), laminin-binding protein (*eno*), elastin binding (*ebpS*), collagen-binding protein (*cna*), fibrinogen-binding protein (*fib*), fibronectin-binding protein (*fnbA* and *fnbB*), biofilm-associated protein (*bap*), serine-aspartate repeat (*sdrC*, *sdrD*, and *sdrE*), and intercellular adhesion (*icaD* and *icaA*) was determined employing PCR assay [14].

### 2.6. Molecular Typing Methods

#### 2.6.1. coa Typing

Multiplex PCR was carried out to determine coagulase (*coa*) types (I-X) based on the method provided by Hirose and coworkers. In this assay, four sets were applied, including (i) set A that identified *coa* types I, II, III, IVa, IVb, Va, and VI; (ii) set B that identified *coa* types VII, VIII, and X; (iii) set C that identified *coa* types IX and Vb; and (iv) set D that differentiated IVa and IVb [15].

#### 2.6.2. MLST

We used the procedure described by the MLST database (https://pubmlst.org/) and the Enright et al. method to characterize HLMUPR isolates; PCR assay was carried out using recommended primers of housekeeping genes (pta, arcC, tpi, aroE, gmk, yqiL, and glp) on the website. Sequence types (STs) were determined by submitting the allelic profile to the online MLST database website.

### 2.7. Statistical Analysis

SPSS Statistics version 22.0 for Windows was used for statistical analysis. In addition, data were analyzed using the chi-square or Fisher's exact tests. *P* values less than 0.05 were deemed statistically significant.

## 3. Results

### 3.1. Antimicrobial Activities

Of the total *S. aureus* isolates, 48 (12.3%) isolates were confirmed as HLMUPR. In our study, HLMUPR isolates were collected from hospitalized patients, 32 (66.7%) patients were male, and 16 patients were female (33.3%) with a median age of 38.6 years, ranging from 15 to 63 years. In terms of sample type, the isolates were obtained from wound (20; 41.7%), abscess (10; 20.8%), blood (9; 18.8%), urine (6; 12.5%), and respiratory tract secretions (3; 6.2%). The HLMUPR isolates accounted for 62.5% and 37.5% of HO (30/48) and CO (18/48) cases. All HLMUPR isolates were resistant to methicillin (MRSA). The distribution of resistance among HO and CO HLMUPR strains to drugs is provided in Table 1.

Present results exhibited resistance of 100% of isolates for PEN, followed by 83.3% for GEN, 77.1% for CIP, 70.8% for ERY, 66.7% for TET, 43.8% for KAN, 41.7% for CLI, 41.7% for AMK, 31.3% for TOB, 29.2% for SXT, 22.9% for RIF, 22.9% for SYN, and 8.3% for FUS. Ten resistance patterns were identified, wherein PEN, GEN, KAN, AMK, TET, ERY, CLI, CIP (31.3%, 15/48), PEN, GEN, TET, TOB, ERY, CIP, SXT (20.8%, 10/48), and PEN, GEN, CIP, RIF, SYN (16.7%, 8/48) were the top three frequently identified patterns. We did not identify any HLMUPR isolates with resistance to tigecycline, linezolid, and vancomycin. According to the data, there is a statistically significant relationship between HLMUPR MRSA and resistance to PEN (*P* value 0.022), GEN (*P* value 0.003), TET (*P* value 0.002), ERY (*P* value 0.006), and CIP (*P* value 0.047). Five isolates (10.4%) were inhibited by 0.5 *μ*g/mL of vancomycin, 12 isolates (25%) by 1 *μ*g/mL, and 31 isolates (64.6%) by 2 *μ*g/mL. Of the 48 HLMUPR strains susceptible to tigecycline, 29 had MIC values of 0.25 *μ*g/ml (60.4%) and 19 had MIC of 0.5 *μ*g/ml (39.6%). A total of 4 HLMUPR isolates (8.3%) were resistant to fusidic acid, of which two had MIC 16 *μ*g/mL and two exhibited MIC titer of 8 *μ*g/mL. Thoroughly, all isolates except one isolate showed resistance to ≥3 classes of antibiotics and considered multidrug resistance (MDR) isolates. Of the total isolates, 20, 12, and 2 isolates showed cMLS_B_, iMLSB, and MS phenotypes accounting for 41.7%, 25%, and 4.2%, respectively. Inducible and constitutive clindamycin resistance rate among HO-HLMUPR strains (14.6% and 27.1%) was higher than CO HLMUPR strains (10.4% and 14.6%). All the HLMUPR strains with MS phenotype were isolated from HO cases (Table 2).

### 3.2. Phenotypic Biofilm Formation

Of the 48 strains of HLMUPR studied, 43 (89.6%) isolates were biofilm producers in varying degrees. Twenty-five (52.1%) isolates were identified as strong biofilm producers, 10 (20.8%) were moderate producers, and eight (16.7%) were found to be weak producers. A statistically significant relationship between resistance to mupirocin at a high level and strong biofilm production (*P* value 0.015) among HLMUPR MRSA isolates was seen.

**Figure 1:**
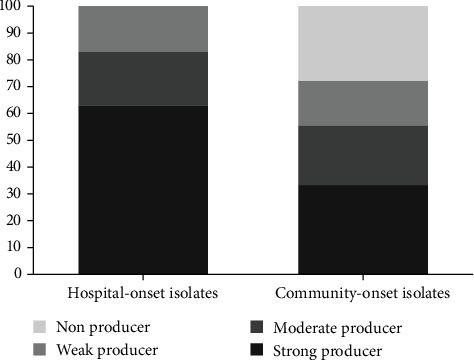
Biofilm formability of isolates based on HO and CO cases.

Five (10.4%) were confirmed as nonbiofilm producer isolates which all belonged to CO cases.  Figure 1 represents the analysis of biofilm formability of isolates based on HO and CO cases.

### 3.3. Toxin Detection

Of 48 HLMUPR MRSA strains, 21 (43.8%) were toxinogenic, with 14 producing *pvl* (29.2%), five *tst* (10.4%), and two *eta* (4.2%). Our analysis indicated a statistically significant relationship between HLMUPR MRSA and toxin carriage (*P* value 0.012).

### 3.4. Genotypic Biofilm Formation

**Figure 2:**
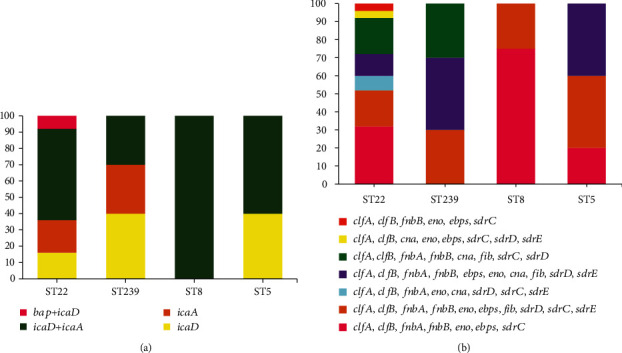
(a) Distribution of biofilm-associated genes in HLMUPR isolates. (b) Distribution of adhesion-associated genes in different molecular types of HLMUPR isolates.

The findings showed that adhesion and biofilm-related genes varied among our isolates. According to our results, among adhesion encoding genes, the *clfA* and *clfB* genes were all detected (100%), *fnbA* was present in 46 strains (95.8%), *fnbB* in 45 (93.8%), *eno* in 40 (83.3%), *sdrC* in 39 (81.3%), *ebps* in 38 (79.2%), *sdrD* in 32 (66.7%), *fib* in 29 (60.4%), *sdrE* in 24 (50%), and *cna* in 20 (41.7%). Regarding biofilm-related genes, *icaD* was present in 10 (20.8%) isolates, *icaA* in 8 (16.7%) isolates, *icaA*+*icaD* in 28 (58.3%) isolates, and *icaD+bap* in two (4.2%) isolates. Seven different biofilm genetic patterns were identified, wherein *clfA*, *clfB*, *fnbA*, *fnbB*, *eno*, *ebps*, *sdrC*, *icaD*, *icaA* (31.3%, 15/48), *clfA*, *clfB*, *fnbA*, *fnbB*, *eno*, *ebps*, *fib*, *icaA*, *icaD*, *sdrD*, *sdrC*, *sdrE* (25%, 12/48), and *clfA*, *clfB*, *fnbA*, *fnbB*, *ebps*, *eno*, *cna*, *fib*, *icaD*, *sdrD*, *sdrE* (18.8%, 9/48) were the top three frequently identified patterns. Biofilm and adhesion-associated genes and their distribution among different STs are provided in Figure 2.

### 3.5. Molecular Typing

In the present work, HLMUPR was classified into three classes, according to the *coa* typing. The predominant *coa* type was III which included 24 isolates (50%), followed by type II (27.1%, 13/48) and type IVa (22.9%, 11/48). According to the MLST, HLMUPR MRSA isolates were assigned to 4 sequences types (STs), including ST22 as the most prevalent (52.1%), followed by ST239 (20.8%), ST8 (16.7%), and ST5 (10.4%). Our analysis clustered tested strains into 3 clonal complexes (CCs), namely, CC22 (52.1%, 25/48), CC8 (37.5%, 18/48), and CC5 (10.4%, 5/48). According to our analysis, nonbiofilm producer isolates belonged to CC8/ST239 (6.3%) and CC/ST8 (4.2%). Regarding biofilm-related genes, the *bap* gene was detected in CC/ST22 isolates. Among the 21-toxinogenic isolates, *pvl* was observed in isolates with CC/ST22 (20.8%, 10/48) and CC/ST8 (8.3%, 4/48). A statistically significant relationship between CC/ST22 and *pvl* carriage (*P* value 0.007), *coa* type III (*P* value 0.041), and strong biofilm formability (*P* value 0.002) was seen. There is also a significant relationship between CC8 and *coa* type III (*P* value 0.047) and strong biofilm formability (*P* value 0.022). All the *tst*-positive isolates belonged to CC8/ST239 (10.4%, 5/48). We found two CC/ST45 strains (4.2%) that carried the *eta* gene. Fusidic acid-resistant isolates belonged to CC/ST45 (*n* = 3) and CC8/ST239 (*n* = 1). Twelve isolates with iMLS_B_ phenotype belonged to CC/ST22 (41.7% (5/12)), CC8/ST239 (33.3% (4/12)), CC/ST8 (16.7%, (2/12)), and CC/ST5 (8.3% (1/12)). Out of 20 strains with cMLS_B_ phenotype, 12 isolates belonged to CC/ST22 (60%), 7 isolates to CC8/ST239 (35%), and 1 isolate to CC/ST8 (5%). Out of two isolates with MS phenotype, one isolate belonged to CC/ST8 and another to CC/ST5.  Table 3 provides data related to the characterization of HLMUPR MRSA strains under study.

## 4. Discussion

The findings showed that 12.3% of *S. aureus* isolates were confirmed as HLMUPR and all carried *mupA* gene. Dadashi et al.'s systematic review and meta-analysis study reported an 8.1% prevalence of HLMUPR MRSA clinical isolates lower than our reported rate. They also reported the prevalence rate of HLMUPR MRSA isolates in Europe, Asia, and the USA, accounting for 8.0%, 12.1%, and 5.9%, respectively [11]. Moreover, Shittu et al. reported that the prevalence of HLMUPR MRSA isolates in Africa ranged between 0.5 and 38% [16]. However, much higher rates were also reported by studies conducted from India (26.1%) [5], the USA (19.3%) [17], and Korea (5.7%) [6]. Reasons for the high resistance rate in Asian countries and our research could be irrational use, various policies in the prescription of these antibiotics, easy access to antibiotics, low cost of the drugs, and spreading of dominant types in these areas. Recent available data has shown there is an increase in the prevalence of *mupA*-positive MRSA isolates. In this connection, Monecke et al. noted a sharp increase in the prevalence of these isolates during the study period from 1.1% in 2000-2015 to 15.9% in 2016 and 17.6% in 2017 [18]. This high prevalence of *mupA* gene is probably because this gene, linked to mupirocin resistance at a high level, is contained within a plasmid whose acquisition was reasonably easy through horizontal gene transfer [10, 11]. In the study, the prevalence of iMLS_B_ was found to be 25% which was near to reported rate from Nepal (21%) [19], higher than those reported in Brazil (7.9%) [20], and lower than the rates reported from Jordan (76.7%) [21]. We observed that 41.7% of isolates had cMLS_B_ phenotype. This was lower than the similar studies carried out in Iran (82.9%) [22] and higher than the rate stated in Nepal [19] (38%) and Greece (20.1%) [23]. It seems that the acquisition of erythromycin resistance in HLMUPR MRSA maybe dynamic and associated with injudicious use of macrolides in patients infected with HLMUPR MRSA isolates [10, 11].

According to the earlier published data, mupirocin-resistant strains are more likely to be resistant to fusidic acid. In our survey, of the forty-eight strains, four isolates were fusidic acid-resistant (8.3%). These results differ from those found in Canada (7%), Ireland (19.9%), Greece (62.4%), and Australia (7%) [24, 25], as well as a study carried out in Iran during four years on 726 tested *S. aureus* isolates, and a low prevalence of resistance to fusidic acid was reported (3%) [26].

In the current research, 29.2% of isolates carried *pvl* gene. Gambian-based research of the prevalence of PVL *S. aureus* in the 11 years reported a rate of 61.4% (180/293) of PVL among *S. aureus* isolates [27]. Different frequency of PVL-positive HLMUPR strains was reported in researches conducted from Iran (27.9%) [28], Ireland (17%) [25], and China (15.1%) [29]. Possible factors involved in the observed high prevalence of *pvl* gene include study population, design, and circulating clones in hospitals and communities.

We detected the *tst*-encoding gene in 10.4% of tested isolates. Nevertheless, Shahini Shams-Abadi et al. showed a relatively high prevalence of *tst* gene among *S. aureus* strains tested (21.3%) [30]. Differences are probably attributed to the origin of isolates, dissemination of specific clones, and sample type.

As mentioned earlier, three different *coa* types were detected in our study. Our data indicated low variability and heterogeneity of *coa* gene. Similarly, a previous study conducted by Omar et al. indicated three *coa* types and ten subtypes [31]. Based on published data by Afrough et al. in Iran [32], six *coa* types with the majority of the pattern I C 1 (21.7%) were detected among *S. aureus* isolates. Consistent with our finding, the results of *coa* typing of 100 S*. aureus* clinical isolates by Salehzadeh et al. noted two genotypes and three subtypes [33]. Contrary to Hirose et al.'s study [15], which displayed a high prevalence of *coa* type II (91.9%), VII (3.9%), and I (1.7%), in our study, *coa* type III was the most predominant *coa* type among *S. aureus* isolates (50%), followed by type II (27.1%) and type IVa (22.9%). Similar results in some studies indicate the predominance of *coa* type III in MRSA isolates [31, 34]. The presence of *coa* type III in the present research and other recent studies suggests that this *coa* type is actively circulating in Tehran province's healthcare setting.

According to the evidence, there is high variability in biofilm-related genes among the MRSA strains and specific genotypes [35]. Earlier studies reported different prevalence rates of biofilm formation among *S. aureus* with data ranging from 43 to 88% [2]. In the present study, the prevalence of biofilm producers was high (89.6%) which was more significant reported in China (66%) [36] and Iran (62.9%) [9]. It is well established that biofilm formation depends on different factors, but it seems that resistance to mupirocin at a high level could affect the accessory gene regulator system involved in the expression of adhesion factors and biofilm induction.

Many studies have noted the critical role of adhesion-related genes in adherence to the host components and producing biofilm. The *clfA*, *clfB*, *fnbA*, and *fnbB* genes are essential for microorganism adhesion and might be related to biofilm formability. In the present study, the complete dominance was observed for *clfA* and *clfB* genes followed by *fnbA* (95.8%) and *fnbB* (93.8%). Similarly, the published results by Yousefi and coworkers in Iran revealed a 69.2% prevalence of biofilm production and the presence of *fnbA*, *clfA*, and *icaA* genes in all examined strains [9]. These rates were comparable to those in other researches [36–40].

Other adhesion-related genes were *eno* and *icaD* (each 83.3%), *sdrC* (81.3%), *ebps* (79.2%), *icaA* (75%), *sdrD* (66.7%), *fib* (60.4%), *cna* (41.7%), and *bap* (4.2%). Nourbakhsh et al. noticed a moderate prevalence of *fnbB* (46.6%), *clfA* (41.4%), *clfB* (44.1%), *fnbA* (38.1%), *ebps* (26.5%), and *cna* (18.3%) genes among their tested isolates [37]. Conversely, in Ghasemian et al.'s study from Iran, the most common biofilm-related genes were *cna* and *ebps* accounted for 78% and 7% of isolates, respectively [38]. In multicenter research performed from 2015 to 2018 from major hospitals in the West Bank-Palestine, out of 248 tested *S. aureus* isolates, 207 (83.5%) possessed the *icaD* gene. In addition, *icaA fnbB*, *cna*, *fib*, *ebps*, *fnbA*, *clfA*/*clfB*, and *eno* genes were found in 16.5, 29, 39.9, 62.2, 76.2, 78.2, 80.2, and 94.8% of isolates, respectively. Azmi and colleagues also showed that none of the strains possessed the *bap* gene [35].

Serine-aspartate repeat (sdr) family proteins have the primary role in the development of *ica*-independent biofilms. Regarding *sdr* genes, *sdrC* and *sdrD* were present in 81.3% and 66.7% of isolates. These values agree with the findings of Uribe-García et al. from Mexico who indicated the presence of *sdrC*, *sdrD*, and *sdrE* in the majority of isolates [8]. The percentages obtained in this study were also similar to those reported in Chen et al.'s study which demonstrated the presence of *sdrC* in the majority of isolates (94.3%) [14]. Earlier data plus the findings of the present study have revealed discrepancies in genetic characteristics of biofilm producers that could likely attribute to the distribution of variants of the genotype of *S. aureus* in a different geographic area.

Some published data documented that the ability of strains of *S. aureus* to produce biofilm has related to the synthesis of polysaccharide intercellular adhesion (PIA), which its production is under the control of the *icaADBC* operon. In this study, a high prevalence of *icaA* (75%) and *icaD* (83.3%) was found, which is in line with the findings of Azmi and colleagues [35] and support those of Sharma and coworkers [39] who reported that *icaA/D* found abundantly among biofilm-producing isolates. Contrarily, Azmi et al. [35] indicated that *icaA* and *icaD* genes were present in 16.5% and 83.5% of strains. These findings suggest that the *ica*-dependent system may not be an absolute mechanism in biofilm production, and these strains perhaps use other systems to produce biofilm.

According to our findings, CC/ST22 was a predominate genotype (52.1%). Forty percent of CC22 isolates were PVL-positive. This finding supports previous results reported by Shore et al. [25], Goudarzi et al. [12], and Monecke et al. [3]. Although biofilm formability and antibiotic resistance profiles in CC/ST22 isolates were found to be varied, resistance to mupirocin and biofilm formability in these strains have been described by several investigators [4, 41].

CC8/ST239 was the second prevailing lineage detected accounting for 20.8% of tested isolates. In line with the present study, ST239 was confirmed as the most predominant genotype in China from 2005 to 2013 in a study conducted by Li et al. [42]. One of the four fusidic acid-resistant MRSA strains belonged to CC8/ST239; this agreed with the findings of Shore et al. from Ireland [25]. Concordant with our study, in a 13-year study, Boswihi et al. in Kuwait displayed fusidic acid-resistant isolates belonged to ST239-MRSA-III that none of them carried *fusB* or *fusC* genes [13]. Inducible clindamycin resistance was noted in 40% of the ST239 isolates. In support of this, Abimanyu and coworkers recently reported the emergence of ST239 MRSA strains with both mupirocin and inducible clindamycin resistance in India [43].

Our analysis confirmed CC/ST8 (ACME+ and PVL+) isolates that resembled the USA300. Earlier researches have noted the resistance to mupirocin and trimethoprim-sulfamethoxazole among USA300 isolates [3, 4, 44, 45], in conformity of results obtained from McDougal et al. in the USA, which identified USA300 clone MRSA isolates harboring *mupA* [44]. We found *mupA* gene in 12.3% of examined isolates. Contrary to a prior study from Iran, which described ST8 S*. aureus* isolates with resistance to vancomycin [45], none of CC/ST8 isolates described in this study was vancomycin-resistant. The emergence of the USA300 in the present study reflects its growing importance as an epidemic clone, which may be due to an import of this clone from Asian or European countries.

In this study, we noted a low prevalence of ST5 (10.4%) with 100% biofilm formability. González-Domínguez and colleagues analyzed 147 MRSA strains isolated from different clinical samples and found that HLMUPR-MRSA isolates belonged to ST125 (97.5%) and ST5 (2.5%) [46]. Present work showed that half of the isolates had a high resistance rate to trimethoprim and fusidic acid. These findings confirmed a study conducted by Shore et al. which indicated resistances to trimethoprim and tetracycline among CC5 isolates at a high level [25].

## 5. Conclusion

Altogether, our study provides evidence for the occurrence of four different lineages of HLMUPR-MRSA in Iran. The high prevalence of CC/ST22 with strong biofilm formation in our hospitals highlights special attention for implementing efficient control protocols and stricter precautions to stop the dissemination of these isolates in both communities to hospitals. There is a need for continuous monitoring of the genotypes of HLMUPR-MRSA isolates to prevent nosocomial outbreaks of these isolates.

## Figures and Tables

**Table 1:** Distribution of resistance among HO and CO HLMUPR MRSA isolated from clinical samples.

Antibiotic	Mupirocin-resistant *S. aureusn* (%)		Total *n* (%)
	Hospital onset	Community onset	
Penicillin	30 (62.5)	18 (37.5)	48 (100)
Gentamicin	24 (60)	16 (40)	40 (83.3)
Kanamycin	13 (61.9)	8 (38.1)	21 (43.8)
Amikacin	14 (70)	6 (30)	20 (41.7)
Tobramycin	8 (53.3)	7 (46.7)	15 (31.3)
Tetracycline	19 (59.4)	13 (40.6)	32 (66.7)
Erythromycin	22 (64.7)	12 (35.3)	34 (70.8)
Clindamycin	13 (65)	7 (35)	20 (41.7)
Ciprofloxacin	22 (59.5)	15 (40.5)	37 (77.1)
Rifampin	7 (63.6)	4 (36.4)	11 (22.9)
Trimethoprim-sulfamethoxazole	9 (64.3)	5 (35.7)	14 (29.2)
Quinupristin-dalfopristin	7 (63.6)	4 (36.4)	11 (22.9)
Fusidic acid	4 (100)	0 (0)	4 (8.3)

**Table 2:** A summary of MDR among samples under study.

Simultaneous resistance to antibiotics	Resistance profile	Resistance pattern^a^	Type of samples^b^ (*n*; % indicated when not 100%)	Number of isolates (%)
Eight	A	PEN, GEN, KAN, AMK, TET, ERY, CLI, CIP	W (6; 40), B (3; 20), A (6; 40)	15 (31.3)

Seven	B	PEN, GEN, TOB, TET, ERY, CIP, SXT	W (7; 70), A (2; 20), U (1; 10)	10 (20.8)
	C	PEN, GEN, KAN, TOB, TET, ERY, CLI	R (1; 20), A (2; 40), U (2; 40)	5 (10.4)

Five	D	PEN, CIP, RIF, SYN, FUS	W (1; 50), B (1; 50)	2 (4.2)
	E	PEN, AMK, ERY, SXT, FUS	B (1)	1 (2.1)
	F	PEN, GEN, CIP, RIF, SYN	W (3; 37.5), U (1; 12.5), B (2; 25), R (2; 25)	8 (16.6)
	G	PEN, GEN, AMK, CIP, RIF	U (1)	1 (2.1)
	H	PEN, KAN, CIP, TET, SYN	U (1)	1 (2.1)

Four	I	PEN, GEN, TET, FUS	B (1)	1 (2.1)
	J	PEN, AMK, ERY, SXT	W (2; 66.7), B (1; 33.3)	3 (6.2)

Without	K	-	W (1)	1 (2.1)

^a^AMK: amikacin; CLI: clindamycin; CIP: ciprofloxacin; ERY: erythromycin; FA: fusidic cid; GEN: gentamicin; KAN: kanamycin; PEN: penicillin; RIF: rifampicin; SYN: quinupristin-dalfopristin; SXT: trimethoprim-sulfamethoxazole; TET: tetracycline; TOB: tobramycin. ^b^W: wound; B: blood; A: abscess; U: urine; R: respiratory tract secretions.

**Table 3:** Characteristics of the HLMUPR MRSA strains isolated from patients.

Clonal complex (CC) (*n*)	Sequence type (*n*)	*coa* type (%)	Antibiotic resistance profile^a^ (%)	Toxin genes (%)	Biofilm status^b^ (%)	Adhesion/biofilm profile^c^ (%)	Type of infection (%)
22	ST22 (25)	III (48), IVa (32), II (20)	A (36), B (36), C (20), F (8)	*pvl* (40)	S (72), M (28)	I (8), II (40), III (20), IV (16), VI (16)	HO (64), CO (36)

8	ST239 (10)	III (70), II (30)	A (50), F (20), I (10), H (10), G (10)	*tst* (50)	S (50), W (20), N (30)	II (50), IV (20), VI (20), VII (10)	HO (60), CO (40)
	ST8 (8)	III (62.5), IVa (37.5)	F (50), J (37.5), A (12.5)	*pvl* (50)	S (25), W (50), N (25)	III (37.5), VI (37.5), V (25)	HO (62.5), CO (37.5)

5	ST5 (5)	II (100)	D (40), E (20), B (20), K (20)	*eta* (40)	M (60), W (40)	V (60), VII (40)	HO (60), CO (40)

^a^Phenotypic profile is shown in Table 2. ^b^S: strong biofilm producer; M: moderate biofilm producer; W: weak biofilm producer; N: no biofilm producer. ^c^Adhesion/biofilm profiles are exhibited in Figure 2.

## Data Availability

All data generated or analyzed during this study are included in this published article.
